# Facteurs pronostiques et survie des leucémies aiguës de l'adulte au Burkina Faso

**DOI:** 10.48327/mtsi.v3i3.2023.409

**Published:** 2023-08-19

**Authors:** Catherine TRAORÉ, Koumpingnin NEBIÉ, Salam SAWADOGO, Adjaratou Fabienne SANOU, Arsène HÉMA, Éléonore KAFANDO

**Affiliations:** 1 Institut supérieur des sciences de la santé (IN.S.SA), Université Nazi Boni, Bobo-Dioulasso, Burkina Faso; 2 Laboratoire d'hématologie, UFR SDS, Université Joseph Ki Zerbo, Ouagadougou, Burkina Faso; 3 Laboratoire d'hématologie, UFR SDS, Université Joseph Ki Zerbo, Ouagadougou, Burkina Faso; 4 Centre hospitalier universitaire de Bogodogo, Ouagadougou, Burkina Faso; 5 Centre hospitalier universitaire Sourô Sanou, Bobo-Dioulasso, Burkina Faso; 6 Centre hospitalier universitaire Yalgado Ouédraogo, Ouagadougou, Burkina Faso

**Keywords:** Pronostic, Survie, Leucémie aiguë, Adulte, Burkina Faso, Afrique subsaharienne, Prognosis, Survival, Acute leukemia, Adult, Burkina Faso, Sub-Saharan Africa

## Abstract

**Introduction/justification:**

Les leucémies aiguës constituent une urgence à la fois diagnostique et thérapeutique. Notre étude a pour but de décrire les facteurs pronostiques et la survie des adultes atteints de leucémies aiguës au Burkina Faso.

**Patients et méthodes:**

Étude transversale, descriptive à collecte de données rétrospective couvrant une période de 4,5 ans (2018-2022) dans deux centres hospitaliers et universitaires du Burkina Faso. Ont été inclus tous les patients de plus de 18 ans hospitalisés pour leucémie aiguë dans ces sites.

**Résultats:**

Au total, 42 cas ont été colligés dont 45% étaient atteints de leucémie aiguë lymphoblastique (LAL) et 43% de leucémie aiguë myéloïde (LAM). Dans 12% des cas, la leucémie aiguë n'était pas classée. Les patients présentaient dans 12% des cas un âge de mauvais pronostic. Les comorbidités étaient présentes chez 14% des patients. L'altération de l'état général était assez constante avec 95% des patients aux stades 3 et 4 de l'OMS. L'hyperleucocytose au diagnostic était présente dans 67% des cas. Le décès en milieu hospitalier était constaté chez 38% des patients. La médiane de survie était de 3 mois. La survie était de 30% à 6 mois et de 0% à 12 mois.

**Conclusion:**

Les leucémies aiguës demeurent dans notre pratique des affections de mauvais pronostic avec une survie courte.

## Abstract (en)

**Introduction:**

Acute leukemia is both a diagnostic and therapeutic emergency. Our study aimed to describe the prognostic factors and survival of adults with acute leukemia in Burkina Faso.

**Patients and methods:**

Cross-sectional descriptive study with retrospective data collection covering a period of 4.5 years (2018-2022) in two university hospitals in Burkina Faso. Were included all patients over 18 years hospitalized for acute leukemia in these sites with a usable medical record.

**Results:**

A total of 42 cases were collected, of which 45% suffered from acute lymphoblastic leukemia and 43% from acute myeloid leukemia. In 12% of cases, acute leukemia was not classified. The average age was 35 ± 15 years, with extremes of 19 and 72 years. 12% of the patients presented an age of poor prognosis. Comorbidities were present in 14% of patients. The deterioration in general condition was fairly constant with 95% of patients at WHO stages 3 and 4. All patients presented with bone marrow failure syndrome and tumor syndrome was found in 45%. Anemia and thrombocytopenia were present in almost all cases. Hyperleukocytosis at diagnosis was present in 28 patients (67%); among them 18 patients (64%) had leukocytes greater than 50 G/L. Death in hospital was found in 38% of patients and loss of sight in 31%. The median survival was 3 months. Survival was 30% at 6 months and 0% at 12 months.

**Conclusion:**

Acute leukemias are in our practice conditions of poor prognosis with a fairly short survival.

## Introduction

Les leucémies aiguës (LA) sont des pathologies malignes très agressives des cellules souches de l'hématopoïèse. Elles constituent une urgence à la fois diagnostique et thérapeutique. L'insuffisance médullaire (avec anémie profonde, syndrome hémorragique et syndrome infectieux) ainsi que la coagulation intravasculaire disséminée, le syndrome de lyse tumorale et le syndrome de leucostase sont des situations d'urgence qui peuvent engager rapidement le pronostic vital du patient. Trois groupes de facteurs affectent le pronostic de la maladie et la survie des patients : facteurs liés au patient (âge au-delà de 60 ans, les antécédents et comorbidités, l'état général), à la maladie (anomalies cytogénétiques et moléculaires, hyperleucocytose) et à la réponse aux traitements [9]. Un traitement précoce et adapté apporte des chances considérables de survie. D'importants progrès ont été réalisés ces deux dernières décennies dans le traitement des LA dans les pays développés. Les découvertes récentes concernant la pathogenèse ont permis le développement de nouvelles approches thérapeutiques pour tous les types de LA et amélioré la survie des patients [35]. Actuellement en Europe, les recommandations d'un groupe d'experts basées surtout sur la cytogénétique et la biologie moléculaire permettent de prédire la survie des patients [10,11,12,19]. En Afrique, les études sur les leucémies aiguës sont limitées et la thématique du pronostic n'est quasiment pas traitée en raison de plateaux techniques insuffisants [13, 14]. Le Burkina Faso, à l'instar des autres pays d'Afrique subsaharienne, ne dispose pas de centres spécialisés de prise en charge des LA. Le traitement spécifique chez l'adulte y reste décevant du fait du manque d'infrastructures adaptées, de l'inaccessibilité des molécules anticancéreuses et de l'insuffisance du support transfusionnel. La cytogénétique, aide diagnostique clé permettant de stratifier les groupes pronostiques, n'est pas accessible à tous. C'est dans un tel contexte que nous avons entrepris le présent travail dans le but de décrire les facteurs pronostiques initiaux et la survie des patients adultes atteints de LA.

## Patients et méthodes

Étude transversale, descriptive à collecte rétrospective des données réalisée du 1^er^ janvier 2018 au 30 juin 2022, soit 4,5 ans. Elle s'est déroulée dans le service d'oncohématologie du Centre hospitalier universitaire (CHU) de Bogodogo à Ouagadougou et dans le service de médecine interne du CHU Sourô Sanou à Bobo-Dioulasso. Ont été inclus les patients âgés d'au moins 18 ans, hospitalisés pour LA *de novo,* ayant un dossier médical exploitable. Était considérée comme LA *de novo,* toute LA non secondaire à un traitement préalable (chimiothérapie ou radiothérapie), à l'évolution d'une hémopathie préexistante, ou sans facteurs de risque retrouvés. Nous avons collecté les données sociodémographiques (âge, sexe), cliniques (motif d'admission, état général, signes cliniques) et biologiques (données de l'hémogramme, myélogramme notamment la blastose médullaire, type cytologique de la leucémie aiguë, classification FAB). Le traitement administré et le devenir du patient ont été collectés. Le diagnostic de LA a été établi à la cytologie avec une blastose médullaire d'au moins 20%. L'anémie était définie par un taux d'hémoglobine inférieur à 12 g/dL, l'hyperleucocytose était évoquée devant une numération des globules blancs supérieure à 10 G/L et la thrombopénie lorsque la numération des plaquettes était inférieure à 150 G/L. La survie globale était calculée à partir de la date du diagnostic jusqu'au décès.

Les données recueillies ont été analysées à l'aide du logiciel Stata 2013. Les résultats ont été exprimés en moyenne ± écart-type et en pourcentage. La survie globale a été estimée en utilisant la méthode de Kaplan-Meier. Une valeur de p inférieure à 0,05 était considérée comme statistiquement significative.

## Résultats

### Caractéristiques générales des patients

Au total, 42 cas étaient colligés sur 4,5 ans dans les deux centres avec une moyenne de recrutement de 8,4 cas par an soit 0,39/100 000 habitants/an. Le sex-ratio (H/F) était de 1,47. L'âge moyen au diagnostic était de 35,8 ± 15 ans, avec des extrêmes de 19 et 72 ans. Dans 86% des cas, les patients étaient hospitalisés par le service des urgences médicales. La pâleur était le motif d'admission le plus fréquent avec 64%. Au moment du diagnostic, tous les patients présentaient un syndrome d'insuffisance médullaire et un syndrome tumoral était retrouvé chez 45% (Tableau I). Le syndrome tumoral était composé de l'association adénopathies et splénomégalie dans 45%, de splénomégalie isolée dans 21%, d'adénopathie isolée dans 16% des cas.

**Tableau I:** Caractéristiques générales des patients atteints de leucémies aiguës, 2018-2022, Burkina Faso (n = 42) Baseline characteristics of patients with acute leukemia, 2018-2022, Burkina Faso (n = 42)

Paramètre	Nombre	Proportion (%)
**Sexe**		
**masculin**	25	60
** féminin**	17	40
**Âge**		
**≤ 20**	6	14
**21 - 30**	12	29
**31 - 40**	12	29
**41 - 50**	5	12
**51 - 60**	2	5
**> 60**	5	12
**Motif de consultation**		
**pâleur**	27	64
**altération de l'état général**	8	19
**fièvre**	4	9
**dyspnée**	2	5
**saignement (gingivorragie)**	1	2
Comorbidités*	6	14
**Insuffisance médullaire**	42	100
**syndrome anémique**	37	88
**syndrome infectieux**	25	59
**syndrome hémorragique**	10	24
**Syndrome tumoral**	19	45
**Hyperleucocytose**	28	67
**Anémie**	41	98
**Thrombopénie**	38	90
**Type cytologique**		
LAM**	18	43
LAL***	19	45
**Non classé**	5	12

* Hépatite virale B (2), hémorroïdes (1), insuffisance cardiaque (1), hémoglobinopathie C (1), kyste pilonidal (1)

** LAM : Leucémie aiguë myéloblastique

*** LAL : Leucémie aiguë lymphoblastique

Le taux moyen des blastes médullaires était de 43% et le nombre moyen de globules blancs était de 86,3 G/L. Une hyperleuco-cytose au diagnostic était présente dans 67% des cas dont 64% avaient des leucocytes supérieurs à 50 G/L. Une anémie et une thrombopénie étaient présentes dans la quasi-totalité des cas. La thrombopénie était sévère (< 20 G/L) dans 34% des cas.

Au total, 45% (19/42) étaient de lignée lymphoïde et 43% (18/42) de lignée myéloïde. Dans 12% des cas, la LA n'était pas classée. La LAL2 est le sous-type le plus fréquemment rencontré (Tableau II).

**Tableau II:** Types cytologiques et sous-types selon la classification franco-américano-britannique (FAB) des leucémies aiguës chez les adultes, 2018-2022, Burkina Faso (n = 42) Cytological types and subtypes according to the French-American-British (FAB) classification of acute leukemia in adults, 2018-2022, Burkina Faso (n = 42)

Type de LA	Sous-types FAB	Nombre	Pourcentage (%)
**LAL**	LAL2	15	36
	LAL3	4	9
**LAM**	LAMO	2	5
	LAM1	4	9
	LAM2	2	5
	LAM4	4	9
LAMS	5	14
**LA non classée**	-	6	12

### Facteurs pronostiques

Les patients présentaient dans 12% des cas un âge de mauvais pronostic (> 60 ans), exclusivement chez les patients LAM. Les comorbidités étaient présentes chez 14% des patients. Une hyperleucocytose supérieure à 50 G/L était notée chez 43% (Tableau III). Aucun résultat de cytogénétique ni de biologie moléculaire n'était disponible pour ces patients.

**Tableau III:** Facteurs pronostiques selon le type cytologique de leucémie aiguë, 2018-2022, Burkina Faso (n = 42) Prognostic factors according to the cytological classification of acute leukemia, 2018-2022, Burkina Faso (n = 42)

Facteurs pronostics	Total n (%)	LAM n (%)	LAL n (%)	LA non classées n (%)
**Facteurs liés au patient**				
**Âge > 60 ans**	5 (12)	5 (12)	0	0
**comorbidités**	6 (14)	4 (9)	2 (5)	0
**performance OMS 3 & 4**	40 (95)	18 (43)	17(40)	5 (12)
**Facteurs liés à la maladie**				
**leucocytose (> 50 G/L)**	18 (43)	8 (19)	7 (17)	3 (7)
**sous-type LAM 7**	0	0	0	0
**cytogénétique**	0	0	0	0

### Traitement et survie des patients

Un traitement symptomatique (composé de la réanimation hématologique, anti-infectieuse et hydro-électrolytique) a été administré chez tous les patients et un traitement palliatif chez 10 patients. Aucun patient n'a reçu de traitement curatif spécifique. Le décès en milieu hospitalier était constaté chez 38% des patients (Tableau IV).

**Tableau IV:** Répartition selon le traitement et le devenir des patients Table IV: Distribution by treatment and outcome of patients

Variables	Modalités	Nombre	Pourcentage
**Traitement**	Symptomatique	42	100
	Palliatif	10	24
	Curatif	0	0
**Devenir**	Décès à l'hôpital	16	38
	Perdu de vue	13	31
	Sorti contre avis médical	11	26
	Transfert	2	5

**Figure 1:**
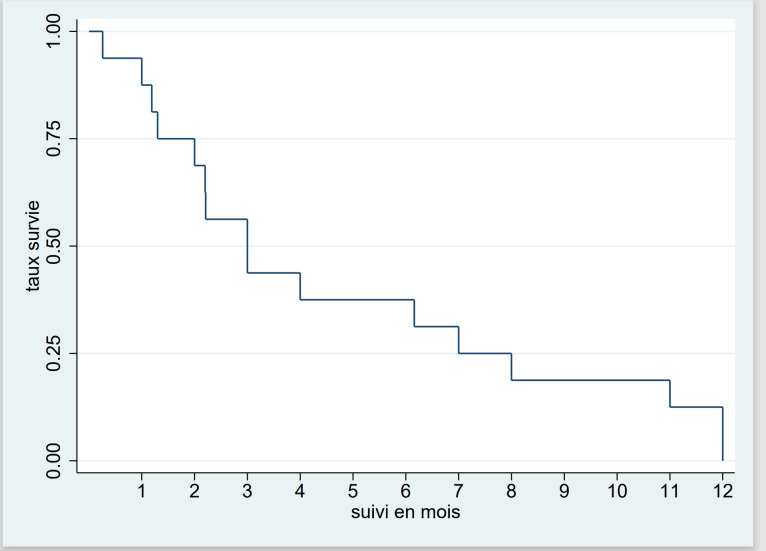
Courbe de Kaplan-Meier de survie globale dans la leucémie aiguë chez l'adulte au Burkina Faso, 2018-2022 Figure 1: Kaplan-Meier curve of overall survival in acute leukemia in adults in Burkina Faso, 2018-2022

La médiane de survie (IQ) était de 3 mois (1,6-7,5). La survie était de 30% à 6 mois et de 0% à 12 mois (Fig. 1).

**Figure 2:**
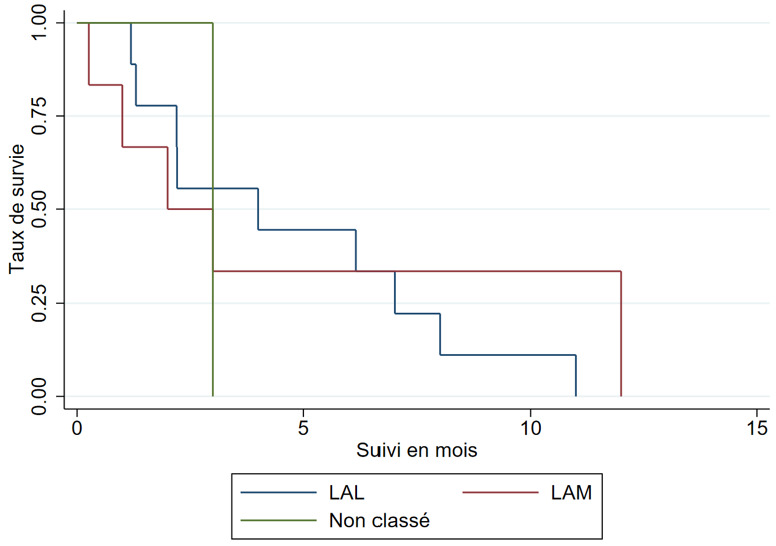
Courbe de Kaplan-Meier de survie selon le type cytologique dans la leucémie aiguë chez l'adulte au Burkina Faso, 2018-2022 Figure 2: Kaplan-Meier curve of survival according to cytological type in acute leukemia in adults in Burkina Faso, 2018-2022

La médiane de survie (IQ) était de 2 mois (1-3) dans les LAM et de 4 mois (2-7) dans les LAL (Fig. 2).

**Figure 3:**
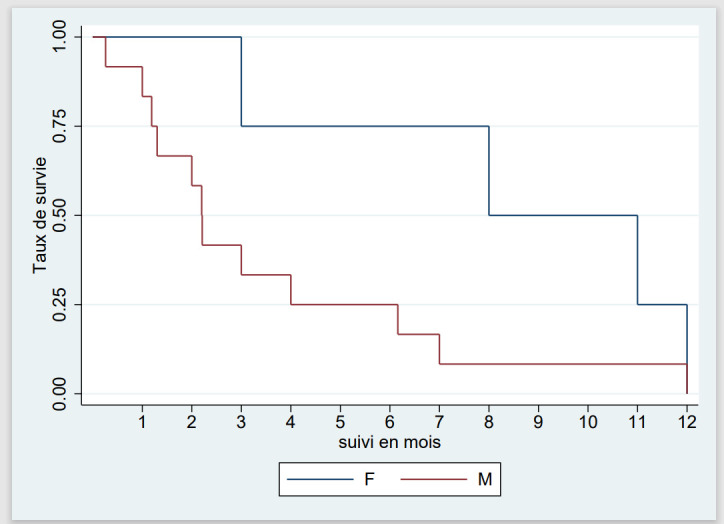
Courbe de Kaplan-Meier de survie selon le sexe dans la leucémie aiguë chez l'adulte au Burkina Faso, 2018-2022 Figure 3: Kaplan-Meier curve of survival according to sex in adult acute leukemia in Burkina Faso, 2018-2022

Chez les patients de sexe féminin, la médiane de survie (IQ) était de 9,5 mois (5-11) contre 2,2 (1-5) mois chez les hommes (Fig. 3).

## Discussion

Notre étude avait pour objectif de décrire les facteurs pronostiques et la survie des patients adultes atteints de leucémie aiguë. Nous avons noté une mortalité hospitalière de 38% et une survie globale de 30% à 6 mois. Cette étude est l'une des rares consacrées à la survie des patients dans la leucémie aiguë dans notre pays. Toutefois, elle comporte des limites relatives à son caractère hospitalier d'une part, et d'autre part à son caractère rétrospectif qui n'a pas permis de disposer de toutes les données à partir des dossiers cliniques. En outre, la non-réalisation des examens de cytogénétique et de biologie moléculaire pour affiner le diagnostic et la non-accessibilité des patients à un traitement spécifique font que notre étude ne prend pas en compte des facteurs pronostiques importants. Ces éléments limiteront la comparabilité de nos résultats à ceux d'autres auteurs.

L'incidence hospitalière était de 8,4 cas par an et de 0,39/100 000 habitants/an. Des résultats similaires ont été rapportés au Cameroun avec 0,36 cas/100 000 habitants [27] et en Algérie avec 0,37 cas/100 000 habitants [6]. Il est probable que l'incidence soit sous-estimée dans les populations africaines compte tenu de l'organisation du système sanitaire où les patients n'ont pas systématiquement accès aux services spécialisés, entraînant une déperdition lors du parcours de la pyramide sanitaire. En outre, le niveau de pauvreté des populations occasionne le recours à des pratiques autres que la médecine conventionnelle.

### Facteurs pronostiques

#### Facteurs pronostiques liés au patient

L'âge semble conditionner le pronostic de manière indépendante des variables biologiques [33]. Les altérations génétiques associées à un âge élevé sont associées à un mauvais pronostic (fréquence élevée des anomalies des chromosomes 5, 7 et 17) [3]. En outre, l'âge modifie la tolérance à la chimiothérapie. Dans notre étude, nous avons observé un âge supérieur à 60 ans dans 12% des cas, exclusivement chez les patients atteints de LAM. Ce résultat est proche de ceux en Corée du Sud (13,7% de patients de plus de 60 ans) [20]. Dans les pays développés, les LAM sont des affections du sujet âgé avec un âge médian de 65 à 70 ans et une incidence qui augmente avec l'âge [5, 16]. L'âge demeure le facteur pronostique le plus important pour la réussite du traitement d'induction des LAM [30]. D'après une étude allemande, la survie globale à 4 ans des patients traités par induction et âgés de moins de 60 ans était de 37%, contre 16% chez les patients de plus de 60 ans [8].

La préexistence de comorbidités aggrave les complications liées au traitement et pose le problème de la stratégie thérapeutique à adopter. Certaines d'entre elles contre-in-diquent la réalisation d'une chimiothérapie. Nos patients présentaient dans 14% des cas une comorbidité. Ce résultat est inférieur à celui de Lerch *et al.* en Suisse qui ont rapporté 62% de comorbidités [24]. Cette faible proportion de notre étude pourrait s'expliquer par la non-exhaustivité du bilan para-clinique.

Outre l'âge du patient et les comorbidités, l'état général doit être pris en compte avant toute décision thérapeutique à visée curative. L'altération de l'état général était constante dans notre étude avec 95% des patients aux stades 3 et 4 de l'OMS. Ce résultat concorde avec celui de Sangaré *et al.* en Côte d'Ivoire qui ont rapporté une altération de l'état général dans 94% des cas [29]. Il est supérieur à ceux d'Allouda *et al.* en Algérie avec 43% des cas [2] et de Lerch en Suisse avec 14% de stades 3 et 4 de l'OMS [24]. Cette différence de proportions peut s'expliquer par la différence du niveau d'organisation des soins, du niveau de scolarisation des populations, de l'existence de couverture sanitaire en Europe contrairement à l'Afrique et du long délai de consultation dans nos contrées.

#### Facteurs pronostiques liés à la maladie

La cytogénétique est un facteur clé dans l'évaluation pronostique des patients atteints de leucémie aiguë. Elle a pour but d'identifier les anomalies chromosomiques afin d'adapter le choix thérapeutique. Elle est systématiquement réalisée dès le diagnostic de LA dans les pays développés où 25 à 30% des LAM sont classées de mauvais pronostic [9, 18]. Un taux de réalisation de la cytogé-nétique de 15% a été rapporté en Algérie, un pays à développement intermédiaire [2]. Dans notre étude comme dans d'autres en Afrique subsaharienne, la cytogénétique n'était pas réalisée, conséquence du manque de plateau technique approprié dans le pays. Les politiques de santé restent prioritairement axées sur la lutte contre les maladies infectieuses, notamment le paludisme, la tuberculose et le VIH/Sida [22]. Par contre, l'épidémiologie des cancers au Burkina Faso est incomplète du fait de l'absence de registre des cancers [26]. Ainsi, l'épidémiologie des LA est hospitalière et probablement sous-estimée, avec une incidence de 3% chez les hommes et 1,6% chez les femmes tous types confondus selon les données anciennes [17]. De plus, les structures adaptées de prise en charge sont inexistantes. La création de centres spécialisés dotés de moyens diagnostiques, pronostiques, thérapeutiques et de suivi nécessite la mobilisation de ressources financières importantes dans un contexte de moyens limités. Les ressources humaines médicales et paramédicales formées à la prise en charge des leucémies aiguës sont insuffisantes. Par conséquent, la réalisation de la cytogénétique pour le pronostic des leucémies des LA est actuellement impossible. Considérant tous ces obstacles, il est urgent d'actualiser l'ampleur des pathologies cancéreuses, en particulier des leucémies aiguës, afin de susciter l'inclusion de leur prise en charge dans les stratégies de la politique sanitaire du pays.

La leucocytose est un signe biologique qui intervient dans l'évaluation pronostique initiale des leucémies. L'hyperleucocytose est un facteur de mauvais pronostic. Une leucocytose supérieure à 50 G/L est un signe de gravité immédiate et expose à un risque élevé de rechute [28]. Elle était présente chez 67% de nos patients. Elle était de 58% dans l'étude de Benzineb *et al.* en Algérie et avait négativement influencé la survie globale des patients avec une différence significative de 0,0001 [6]. En Iran, Allahyari *et al.* ont rapporté une leucocytose moyenne de 29,9 G/L avec un taux de survie de 49% des patients présentant moins de 20 G/L, contre 26% pour ceux ayant plus de 20 G/L et une différence significative de 0,006 [1]. En l'absence de couverture sanitaire et de centres spécialisés au Burkina, les malades sont vus tardivement en consultation, à des stades plus avancés avec une masse tumorale plus importante.

Les sous-types cytologiques M6 et M7 constituent également des facteurs de mauvais pronostic des LAM [18]. Ces deux sous-types cytologiques n'étaient pas retrouvés chez nos patients. Cela pourrait être dû d'une part à la rareté de ces sous-types, et d'autre part à la faible taille de notre échantillon. Braham *et al.* en Tunisie rapportent sur 195 cas de LA, 1% de sous-type M7 et 7% de M6 [7]; Lerch en Suisse rapporte sur 128 cas, 5% de M6 et aucun cas de M7 [24].

#### Facteurs pronostiques liés au traitement

Le traitement administré était essentiellement symptomatique et quelquefois palliatif. Aucune chimiothérapie à visée curative n'a été administrée chez nos patients. Des études réalisées dans les situations similaires de ressources limitées au Mali [31], en Inde [25] et au Burkina [15] ont rapporté l'éventualité des traitements spécifiques avec un impact positif sur la survie des patients. Toutefois, ces études ont porté sur des enfants de 1 à 15 ans qui souffraient de leucémies aiguës lymphoblastiques. Ce type de LA présente peu ou pas de comorbidités, un facteur favorable au pronostic du patient. Pour ces cas du Mali et du Burkina Faso, la prise en charge des LAL est subventionnée par le Groupe franco-africain d'oncologie pédiatrique (GFAOP) facilitant l'accès aux médicaments antimitotiques, avec la mise sous traitement dès que le diagnostic est posé. Ce soutien par un organisme indépendant n'est pas étendu aux adultes.

Le traitement de la LA, particulièrement chez l'adulte, requiert des protocoles de chimiothérapie cytotoxique et myélo-suppressive dont la manipulation n'est pas aisée. Ils peuvent entraîner des complications (infections, anémie, risque hémorragique, etc.) [23, 24]. Leur emploi demande une formation des personnels de santé en particulier pour gérer les aplasies médullaires.

#### Survie

Nous avons enregistré 38% de décès en milieu hospitalier. La comparaison de la léta-lité africaine avec celle des études des pays développés est difficile compte tenu de l'écart des moyens de prise en charge. Dans les pays développés, le diagnostic est précis, la classification pronostique est systématique, les moyens de traitement curatif sont accessibles et le traitement est réalisé dans des centres spécialisés. Dans nos contrées, les services d'hématologie sont rares et les centres de prise en charge des leucémies aiguës sont inexistants, les spécialistes sont peu nombreux et le plateau technique est insuffisant. Nous avons observé un taux de perdus de vue de 31%. Ce résultat est superposable à celui de Togo *et al.* au Mali qui ont rapporté 26% [32]. Les perdus de vue demeurent un problème majeur dans les services de traitement des cancers en Afrique et dans tous les pays en développement [21]. La médiane de survie de nos patients était de 3 mois et la survie à 12 mois était nulle. Ce résultat est comparable à celui d'Atiméré *et al.* en Côte d'Ivoire qui ont rapporté une médiane de survie à 90 jours et une survie à 12 mois de 10% [4]. Le faible taux de survie en Afrique subsaharienne est dû à plusieurs facteurs dont le retard à la consultation, l'absence de plateaux techniques appropriés, l'indisponibilité et l'inaccessibilité financière des médicaments anticancéreux, l'insuffisance du support transfusionnel entre autres. La LA non classée sur le plan cytologique était rapidement fatale avec une probabilité de survie nulle à 3 mois, alors que celle de la LAM et de la LAL était respectivement de 30% et de 55%. Nous avons par ailleurs noté une survie plus élevée chez les femmes (médiane de 9,5 contre 2,2 mois). Dans la littérature, des constats similaires ont été faits. Toutefois, les explications sur les différences hormonales avancées ne remportent pas l'unanimité [34].

## Conclusion

Les leucémies aiguës demeurent dans notre pratique des affections de mauvais pronostic avec une survie courte. Pour améliorer la prise en charge et la survie des patients dans nos contrées, il est impératif de doter les structures sanitaires des moyens nécessaires pour un diagnostic précis (myélogramme et immunophénotypage) et une meilleure classification pronostique (cytogénétique et biologie moléculaire). Des moyens logistiques adéquats, l'accès aux anticancéreux à moindre coût, aux produits sanguins labiles et la formation du personnel à la prise en charge sont la clé de la réussite.

## CONTRIBUTION DES AUTEURS

C. Traoré : initiation, collecte, rédaction

K. Nebié : analyse, lecture

S. Sawadogo : analyse, lecture

A. F. Sanou : collecte des données

A. Héma : analyse

É. Kafando : lecture

## LIENS D'INTÉRÊTS

Les auteurs ne déclarent aucun lien et aucun conflit d'intérêts.
